# Hydrogen Sulfide Alleviates Acute Myocardial Ischemia Injury by Modulating Autophagy and Inflammation Response under Oxidative Stress

**DOI:** 10.1155/2018/3402809

**Published:** 2018-08-01

**Authors:** Ya-dan Bai, Yu-rong Yang, Xue-pan Mu, Ge Lin, You-ping Wang, Sheng Jin, Ying Chen, Ming-jie Wang, Yi-chun Zhu

**Affiliations:** ^1^Shanghai Key Laboratory of Bioactive Small Molecules, Shanghai Key Laboratory of Clinical Geriatric Medicine, Department of Physiology and Pathophysiology, School of Basic Medical Sciences, Fudan University, Shanghai 200032, China; ^2^Key Laboratory of Molecular Virology and Immunology, Vaccine Center, Institute Pasteur of Shanghai, Chinese Academy of Sciences, Shanghai 200031, China; ^3^Department of Physiology, Hebei Medical University, Hebei 050017, China

## Abstract

This study aims to investigate the influence of excessive oxidative stress on cardiac injury during acute myocardial ischemia (AMI), with a focus on apoptosis, autophagy, and inflammatory cell infiltration, and to detect the role of hydrogen sulfide (H_2_S) in this process. We found that SOD1 knockout (KO) mice showed excessive oxidative stress and exacerbated myocardium injury after AMI. Increased apoptosis and inflammation response in the ischemic myocardium contribute to this deterioration, whereas enhanced autophagy plays a protective role. Myocardial inflammation after AMI was much more severe in SOD1 KO mice than in wild-type mice. Pretreatment with the H_2_S donor NaHS reduced autophagy and apoptosis levels in the ischemic myocardium and alleviated the regional inflammation response in the cardiac tissues of SOD1 KO mice. Moreover, autophagy and apoptosis levels were significantly enhanced in SOD1 knockdown primary neonatal rat cardiomyocytes (NRCMs) under glucose deprivation. Pretreatment with NaHS can partially inhibit this elevation. Taken together, we found that excessive oxidative stress can aggravate cardiac injury during AMI. Exogenous H_2_S can alleviate cardiac injury during AMI by reducing apoptosis and inflammation response in heart tissues under oxidative stress.

## 1. Introduction

Acute myocardial ischemia (AMI) induced by coronary artery occlusion is one of the leading causes of cardiovascular morbidity and mortality worldwide [1–3]. Acute cardiac ischemia is a multifactorial disease that mainly results in dysfunction of mitochondrial energy metabolism, followed by the initiation of myocardial injury [4, 5]. The main characteristic of impaired mitochondrial metabolism is the production of excessive reactive oxygen species (ROS), including hydrogen peroxide, superoxide, peroxynitrite, and hydroxyl radicals [6–8], which are mainly generated from mitochondria [9]. While low levels of ROS are essential for cellular signaling transduction, massive ROS induces apoptosis and necrosis by oxidative stress. Thus, ROS scavenging by antioxidants is necessary for cell survival. Antioxidant defenses involve enzymes such as superoxide dismutases (SOD), catalases (CAT) and glutathione peroxidases (GPx), and nonenzymatic antioxidants such as vitamins and glutathione (GSH) [10]. As the major antioxidant enzymes, SODs such as Cu/Zn-SOD, Mn-SOD, and EC-SOD (extracellular SOD) play crucial roles in scavenging ROS [11]. Homozygous (−/−) Cu/Zn-SOD (SOD1) KO mice exhibit high levels of oxidative stress [12]. Oxidative stress was induced, and the activity of antioxidant enzymes was decreased in patients suffering from AMI in clinical research [13–15]. However, it is not clear whether people with high ROS levels are susceptible to AMI. In contrast, overexpression of SOD isoforms protects cardiomyocytes from ischemia-reperfusion injury in mice [16, 17]. However, the mechanism of how oxidative stress in SOD1-deficient mice affects cardiac function during AMI *in vivo* is not fully demonstrated.

Autophagy is an intracellular lysosomal degradation pathway [18] which is responsible for the degradation of intracellular proteins and organelles. Autophagy includes three main types: macroautophagy, microautophagy, and chaperone-mediated autophagy [19]. Macroautophagy is the most extensively investigated autophagy, and this study focuses on this type of autophagy and simply refers to it as autophagy hereafter. Macroautophagy is characterized by the formation of autophagosomes which sequester cytoplasmic components and ultimately fuses with lysosomes, where engulfed cargo is degraded [20]. Under physiological conditions, autophagy is important for maintaining cellular homeostasis and keeping cells healthy [21]. Dysregulated autophagy results in various diseases such as infection, neurodegenerative disease, and tumorigenesis [22–25]. It is reported in the literature that autophagy is upregulated in various heart diseases, including acute myocardial ischemia [26–28]. Excessive autophagy can lead to programmed cell death [29]. Dysregulated autophagy and cell death are both involved in myocardial infarction. Myocardial infarction activates innate immune pathways that trigger an intense inflammatory reaction [30]. The proinflammatory chemokines and cytokines are markedly upregulated and contribute to the recruitment and incorporation of inflammatory cells in the infarcted area [31]. The recruited inflammatory cells phagocytose dead cells and matrix debris [25]. Then, a reparative response is followed by resolution of inflammation, myofibroblast proliferation, and formation of a collagen-based scar [32–34]. Taken together, autophagy, cell death, and inflammatory response are important components of cardiac injury and repair in AMI.

Hydrogen sulfide (H_2_S) is a newly found gaseous signaling molecule playing important roles in various physiological and pathophysiological processes. Endogenous H_2_S is produced by catalyzing the substrate L-cysteine by cystathionine *γ*-lyase (CSE), cystathionine-*β*-synthase (CBS), and 3-mercaptopyruvate sulfurtransferase (3-MST). Previous studies have found that H_2_S played vital roles in vascular relaxation by opening the K_ATP_ channels [35, 36], promoting angiogenesis through targeting the disulfide bond-containing receptor proteins [37–39], inhibiting oxidative stress [40], and protecting cardiomyocytes from myocardial ischemia-reperfusion injury [41, 42].

In the present study, we focus on investigating how excessive oxidative stress affects cardiac injury during AMI and the role of exogenous H_2_S in an AMI mouse model with impaired antioxidant defenses.

## 2. Materials and Methods

### 2.1. Acute Myocardial Ischemia Mouse Model and NaHS Administration *In Vivo*

Animal experiments were conducted according to the Guide for the Care and Use of Laboratory Animals of the National Institutes of Health (NIH) of the United States and approved by the ethics committee for the experimental research, Shanghai Medical College, Fudan University. All experiments were performed with 8–12-week-old adult mice. Heterozygous Cu/Zn SOD (SOD1) KO mice (stock number: 002972) were purchased from Jackson Laboratory. Homozygous animals were used in the experiments, while heterozygous animals were used for breeding.

Mice were anesthetized with isoflurane (2%) and ventilated via tracheal intubation with a rodent ventilator (Kent Scientific Corporation, Torrington, CT, USA). The chest was opened, and AMI was achieved by occluding the anterior descending branch of the left coronary artery. Finally, the chest wall was closed. The sham-operated animals experienced the same surgery without ligation of the coronary artery. The hearts were harvested at certain time points.

For mice receiving NaHS pretreatment, 50 *μ*mol/kg NaHS was administered 30 min before AMI surgery by intraperitoneal injection. Saline was used as vehicle control.

### 2.2. Determination of Infarct Size

2,3,5-Triphenyltetrazolium chloride (TTC, Sigma, St. Louis, MO, USA) staining was used to measure the infarct size. Mice were sacrificed 2 h after MI surgery. The heart tissues were excised and cut into 1–2 mm slices. The slices were incubated with 1% TTC at 37°C for 30 min in the dark and fixed in 4% paraformaldehyde. Finally, images were photographed and analyzed by ImageJ software.

### 2.3. Primary Culture of Neonatal Rat Ventricular Cardiomyocytes and NaHS Treatment

Primary neonatal rat cardiomyocytes (NRCMs) were isolated from 1-day-old Sprague-Dawley rats as described previously [43]. The experimental protocols were approved by the ethics committee for the experimental research, Shanghai Medical College, Fudan University. Briefly, the dissected hearts were washed three times with cold phosphate-buffered saline (PBS), minced into 1 mm^3^ pieces, and digested with 0.125% trypsin several times. After digestion, cardiomyocytes were purified by differential attachment and cultured in Dulbecco's modified Eagle medium/F-12 (DMEM/F12) containing 10% fetal bovine serum, 100 U/mL penicillin, and 100 *μ*g/mL streptomycin. 5-Bromo-2-deoxyuridine (Brdu) (0.1 mM) was added during the first 48 h to prevent the proliferation of noncardiomyocytes. For glucose deprivation experiments, cardiomyocytes were washed three times with PBS and incubated with glucose-free DMEM (Invitrogen, Carlsbad, CA, USA) containing no serum. NRCMs were treated with NaHS at a concentration of 50 *μ*M 30 min before glucose deprivation.

### 2.4. Detection of ROS Levels

ROS levels were detected with dihydroethidium (DHE, Sigma-Aldrich, St. Louis, MO, USA). For *in vivo* assays, the heart tissues were excised, embedded in optimal cutting temperature compound (O.C.T. Compound, Sakura, Torrance, California, USA), and then cut into sections (6 *μ*m). The sections were incubated with DHE (10 *μ*M) for 30 min in the dark. Finally, images were captured by a fluorescence microscope (Leica, Wetzlar, Germany). For *in vitro* assays, NRCMs were incubated with DHE (10 *μ*M) for 30 min at 37°C in the dark and detected by flow cytometry.

### 2.5. Western Blot Assay

Heart tissues or primary cardiomyocytes were lysed with lysis buffer (containing 50 mM Tris-HCl (pH 7.4), 1 mM EDTA (pH 8.0), 150 mM NaCl, 1% Triton-100, 0.1% SDS, and 0.25% DOC) supplemented with protease inhibitor cocktails (PIC, Roche, Basel, Switzerland) and phosphatase inhibitor cocktails (Roche, Basel, Switzerland). The protein concentration was determined with a BCA kit (Shen Neng Bo Cai, Shanghai, China). Proteins were separated by sodium dodecyl sulfate-polyacrylamide gels (SDS-PAGE) and transferred to a polyvinylidene difluoride (PVDF) membrane (Millipore, Bedford, MA, USA). After being blocked with 5% BSA, PVDF membranes with separated proteins were incubated with primary antibodies and horseradish peroxidase-conjugated secondary antibodies. Primary antibodies used in the present study included anti-LC3 (NB100-2220, Novus, Littleton, CO, USA), anti-ribosomal S6 kinase (S6K) (2708, Cell Signaling, Danvers, MA, USA), antiphosphorylated S6K (Thr-389) (9205, Cell Signaling, Danvers, MA, USA), antiphosphorylated AMPK (Thr-172) (2535, Cell Signaling, Danvers, MA, USA), anti-ATG5 (12994, Cell Signaling, Danvers, MA, USA), anti-Bax (50599-2-Ig, Proteintech, Rosemont, IL, USA), anti-Bcl2 (12789-1-AP, Proteintech, Rosemont, IL, USA), and anti-GAPDH (60004-1-Ig, Proteintech, Rosemont, IL, USA). Chemiluminescent signals were developed by the addition of the enhanced chemiluminescence (ECL) reagents (Bio-Rad, Hercules, CA, USA). Intensities of protein bands were quantified by ImageJ software.

### 2.6. Terminal Deoxynucleotidyl Transferase- (TdT-) Mediated dUTP Nick End Labeling Assay (TUNEL Assay)

Cleavage of genomic DNA during apoptosis may yield DNA strand breaks, which can be identified by labeling free 3′-OH DNA fragments with modified nucleotides by TdT. In this study, the In Situ Cell Death Detection Kit (Roche, Basel, Switzerland) was used to detect apoptosis. Briefly, the paraffin-embedded heart tissues were sectioned at 6 *μ*m thickness. After dewaxing, the tissue sections were digested with proteinase K and then incubated with TUNEL reaction mixture buffer at 37°C for 60 min in the dark. Then, tissue sections were stained with Fluoromount-G™ with DAPI (eBioscience, San Diego, CA, USA). At last, images were captured by a fluorescence microscope (Leica, Wetzlar, Germany).

### 2.7. siRNA-Mediated SOD1 Knockdown in NRCMs

The designed SOD1-targeting siRNA sequence was 5′-GGTGGTCCACGAGAAACAAGA-3′. The SOD1-targeting siRNA sequence and the negative control siRNA (siNC) were synthesized by Biotend (Shanghai, China). The Atg5-targeting siRNA sequences were designed and synthesized by Biotend (Shanghai, China). After culturing for 48 h, NRCMs were transfected with 40 pmol siRNA per 6-well plate using Lipofectamine RNAiMAX (Invitrogen, Carlsbad, CA, USA) per the manufacturer's instructions. Briefly, siRNA and Lipofectamine RNAiMAX were diluted in Opti-MEM, mixed, and incubated at room temperature for 5 min. Then, the siRNA-Lipofectamine RNAiMAX mixture was added to cells in the medium. Media were replaced with fresh DMEM/F12 complete medium after 6 h incubation. Experiments were performed 48 h after transfection. The knockdown efficiency was detected by Western blotting.

### 2.8. Annexin V-FITC/Propidium Iodide Staining

Apoptosis in NRCMs was measured using the Annexin V-FITC apoptosis detection kit (BioLegend, San Diego, CA, USA) according to the manufacturer's instructions. The NRCMs were harvested by trypsinization and washed twice with PBS and once with binding buffer. Then, the NRCMs were stained with Annexin V and propidium iodide (PI) at 4°C for 15 min. Finally, the NRCMs were resuspended with binding buffer and analyzed by flow cytometry.

### 2.9. Detection of Immune Cells

Two days after AMI, heart tissues and whole blood were isolated from sacrificed mice. After washing several times in PBS, the heart tissues were minced into pieces and digested with digestion buffer containing 1 mg/mL collagenase IV (Sigma, St. Louis, MO, USA) and 20 *μ*g/mL DNase I (Solarbio, Beijing, China) at 37°C for 1 h. Whole blood samples were incubated with red blood cell lysis buffer (eBioscience, San Diego, CA, USA) at room temperature for 10 min to remove erythrocytes. After washing twice, single cells were resuspended in MACS buffer (containing 25 mM EDTA and 1% fetal calf serum in PBS). For cell labeling, fresh cells were incubated with primary antibody on ice using the following antibodies: BV421-labeled anti-CD3 (562600, BD Biosciences, San Jose, CA), APCcy7-labeled anti-CD19 (50-245-965, eBioscience, San Diego, CA, USA), PE-labeled Ly6G (CD183) (108408, BioLegend, San Diego, CA, USA), APC-labeled anti-CD11b (17-0112-82, eBioscience, San Diego, CA, USA), and FITC-labeled Ly6C (11-5931-81, eBioscience, San Diego, CA, USA). The cells were washed twice in MACS buffer and immediately analyzed on a BD LSRFortessa™ cell analyzer.

### 2.10. Immunohistochemical Analysis of Heart Tissues

The paraffin-embedded sections of heart tissues (6 *μ*m) were dewaxed and retrieved by a pressure cooker for 12 min in citrate (pH = 6.0). Then, the sections were incubated with CD11b antibody (ab133357, Abcam, Cambridge, UK) at room temperature for 2 h and subsequently incubated with the horseradish peroxidase- (HRP-) conjugated secondary antibody and the diaminobenzidine substrate. Finally, the images were acquired by a light microscope (Leica, Wetzlar, Germany).

### 2.11. Measurement of Autophagy Flux

To measure autophagy flux *in vivo*, mice were administered 40 mg/kg chloroquine (CQ) by intraperitoneal injection 1 h before AMI. Two hours after surgery, the heart tissues were harvested and LC3 protein expression was examined by Western blotting. For *in vitro* assays, cells were pretreated with CQ (10 *μ*M) for 4 h before harvesting for LC3 immunoblotting.

*In vitro* autophagy flux was also studied using a mCherry-GFP-LC3 probe. The mCherry-GFP-LC3 adenovirus was purchased from Beyotime (Shanghai, China). After 48 h of siRNA transfection, NRCMs were transfected with mCherry-GFP-LC3 adenovirus at a multiplicity of infection (MOI) of 40. Two days later, fluorescent signals were examined by a confocal microscope (Zeiss, Jena, Germany). The yellow and red LC3 puncta were manually counted.

### 2.12. Transmission Electron Microscopy (TEM)

The mice were sacrificed by injecting an overdose of anesthetic, and the heart tissues were quickly removed and washed in PBS several times. The heart tissues were cut into 1 mm transverse sections and immersed in 2% glutaraldehyde overnight. Then, the sections were immersed in 1% osmium tetroxide for 2 h, dehydrated in graded ethanol, and embedded in epoxy resin. Ultrathin sections (60–70 nm) were obtained, poststained with uranyl acetate and lead citrate, and examined using a TEM (Tenai G2 Spirit; Hillsboro, OR, USA).

### 2.13. Enzyme-Linked Immunosorbent Assay (ELISA)

IL-6 and TNF-*α* levels were measured by standard sandwich ELISA protocol from heart homogenates and serum. IL-6 was detected using a rat anti-mouse IL-6 monoclonal antibody (504502, BioLegend, San Diego, CA, USA) for capture and biotinylated rat anti-mouse IL-6 monoclonal antibody (504602, BioLegend, San Diego, CA, USA) for detection. TNF-*α* was measured using a rat anti-mouse TNF-*α* antibody (14-8321, eBioscience, San Diego, CA, USA) for capture and biotinylated rabbit anti-mouse TNF-*α* polyclonal antibody (14-7423, eBioscience, San Diego, CA, USA) for detection.

### 2.14. Statistical Analysis

Quantitative values are presented as the mean ± SE (standard error). The protein levels were normalized to the mean of the control data in each set of experiments. The statistical comparisons among groups were performed by one-way ANOVA. Paired data were evaluated by Student's *t*-test. In all cases, a *p* value of <0.05 was considered significant.

## 3. Results

### 3.1. Cardiac Injury Was Enhanced in SOD1 KO Mice Compared to That in WT Mice after AMI

**Figure 1:**
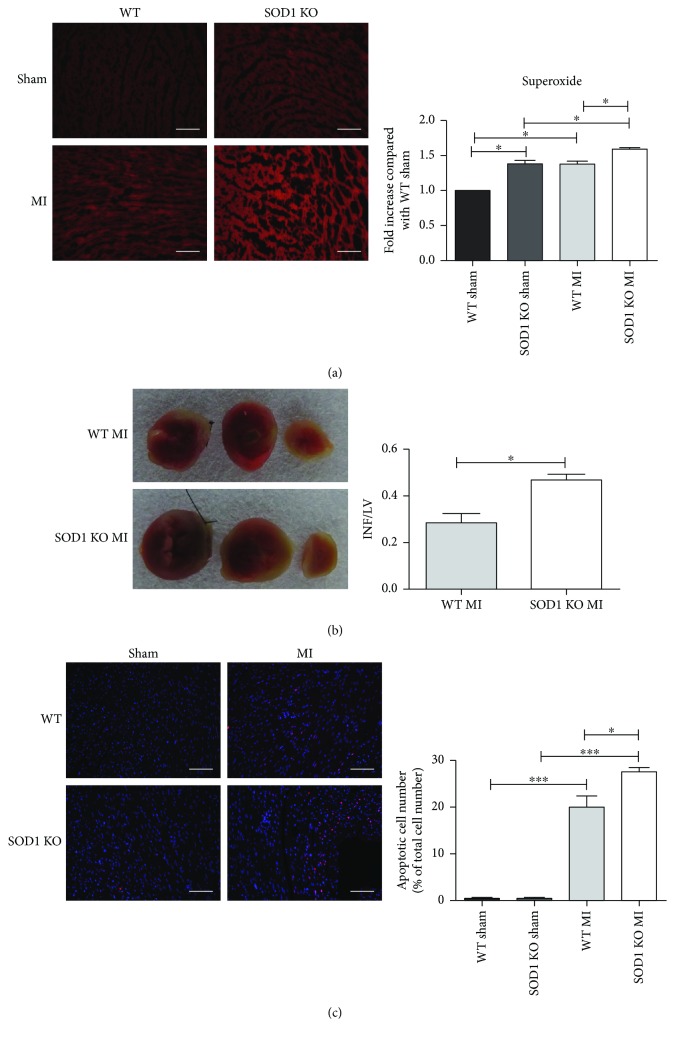
SOD1 KO mice showed enhanced ischemic injury. (a) Superoxide levels in heart tissues were detected by DHE staining. Representative images and quantitative analysis are shown. Scale bar = 50 *μ*m. (b) Representative images and quantitative analysis of the infarcted area. The white area represents INF. LV: left ventricle; INF: infarct area. (c) Apoptotic cells in the myocardium were detected by TUNEL staining. Representative images and quantitative analysis were shown. Scale bar = 50 *μ*m. Values are mean ± SE, *n* = 6 in each group. ^∗^ *p* < 0.05 and ^∗∗∗^ *p* < 0.001.

**Figure 2:**
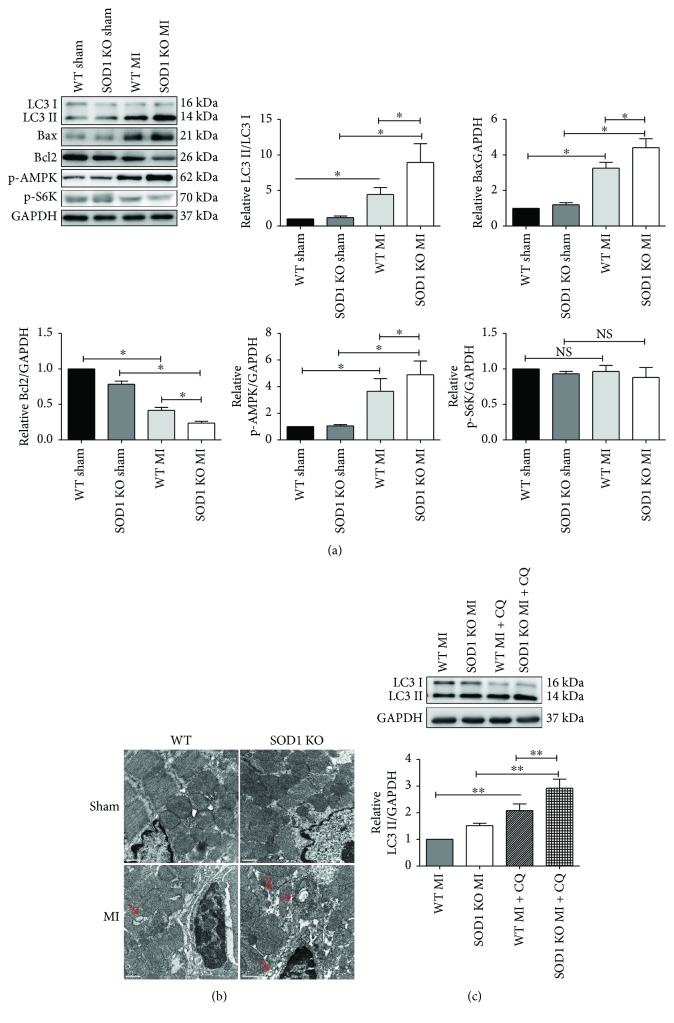
SOD1 KO mice showed enhanced autophagy levels in ischemic myocardium. (a) Representative blots of LC3II/LC3I, Bax, Bcl2, and S6K phosphorylation and AMPK phosphorylation and quantitative analysis of LC3II/LC3I, Bax, Bcl2, p-S6K, and p-AMPK. (b) Representative images of TEM results are shown. Red arrows indicate autophagic vacuoles. Scale bar = 0.5 *μ*m. (c) Myocardium LC3 detection with the presence of 40 mg/kg chloroquine. Representative blots and quantitative analysis of LC3II/GAPDH are shown. Values are mean ± SE, *n* = 6 in each group. ^∗^ *p* < 0.05 and ^∗∗^ *p* < 0.01. NS: no significance.

To investigate the effect of oxidative stress on cardiac injury after AMI, WT and SOD1^−/−^ (SOD1 KO) mice were used. First, superoxide level was measured by DHE staining. As shown in Figure 1(a), the superoxide level was much higher in the myocardium of SOD1^−/−^ mice than that of WT mice. The superoxide levels in the ischemic myocardium of MI groups were significantly higher than those in sham-operated groups. The superoxide level in the ischemic myocardium of SOD1^−/−^ mice was much higher than that of WT mice. To detect cell death in heart tissues, TTC staining and TUNEL staining were performed. TTC staining showed that the infarct area in SOD1^−/−^ AMI mice was significantly higher compared to that in WT AMI mice (Figure 1(b)). The TUNEL assay showed that the percentage of apoptotic cells was significantly higher in AMI mice compared to that in sham-operated controls in both WT and SOD1^−/−^ mice, while the number of apoptotic cells was further increased in SOD1^−/−^ AMI mice than that in WT AMI mice (Figure 1(c)). The Western blotting results showed that the expression of Bax was increased and Bcl2 was decreased in AMI heart tissues in both WT and SOD1^−/−^ mice compared to those in sham-operated groups. The expression of Bax was increased further, and Bcl2 was decreased further in the heart tissues by SOD1 knockout (Figure 2(a)).

Autophagy plays important roles in the heart after AMI [44]. Therefore, autophagy was examined by Western blotting LC3 protein levels and electron microscopy (TEM) of autophagosomes and autolysosomes. The results showed that the ratio of LC3II/LC3I increased significantly in heart tissues subjected to AMI compared to that in the sham-operated groups. The ratio of LC3II/LC3I increased further in the ischemic myocardium in SOD1 KO mice compared to that in WT mice (Figure 2(a)). The AMPK-mTOR pathway is recognized as a crucial regulator of autophagy [26, 45]. Thus, protein analysis was conducted to detect the activity of AMPK and mTOR. Thr172 phosphorylation of AMPK was increased significantly after AMI, while the phosphorylation of S6K, a substrate of mTOR, was not induced. AMPK activity was induced further in the ischemic myocardium in SOD1 KO mice compared with WT mice (Figure 2(a)). The TEM results showed that the number of autophagic vacuoles (AVs) was increased in heart tissues from mice subjected to AMI, and the number of AVs in the ischemic myocardium was increased further in SOD1^−/−^ mice compared to that in WT mice (Figure 2(b)). The accumulation of LC3-II can result from several different aspects, such as interruption of the autophagosome-lysosome fusion step and inhibition of the lysosome-mediated proteolysis. Therefore, the autophagy flux was detected by adding chloroquine (CQ) to raise the lysosomal pH and thus inhibit lysosome-mediated proteolysis. The results showed that LC3-II protein level was increased by the addition of CQ (Figure 2(c)). These results indicated that autophagy was upregulated by AMI, which was accompanied by AMPK activation. Additionally, autophagy was increased further in the ischemic heart tissues of SOD1 KO mice.

**Figure 3:**
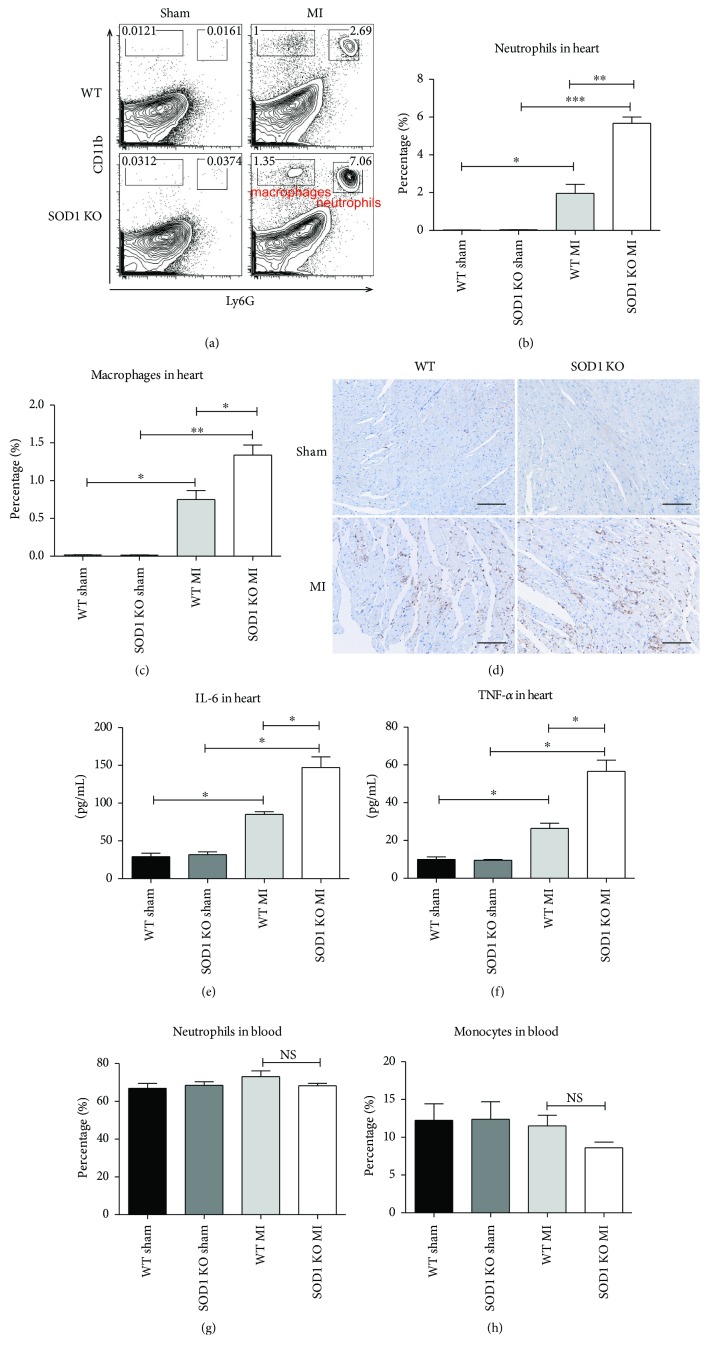
SOD1 KO mice subjected to AMI showed severe inflammation response in the ischemic myocardium. (a) Representative FACS results of inflammatory cell counting from heart samples. (b–c) Quantitative analysis of neutrophils (b) and macrophages (c) in heart tissues. (d) Immunohistochemistry of CD11b (marker of both neutrophils and macrophages). Positive staining is shown in brown. Scale bar = 50 *μ*m. (e–f) Quantitative analysis of inflammatory cytokines IL-6 (e) and TNF-*α* (f) in heart tissues. (g–h) Quantitative analysis of neutrophils (g) and monocytes (h) in blood samples. Values are mean ± SE, *n* = 6 in each group. ^∗^ *p* < 0.05, ^∗∗^ *p* < 0.01, and ^∗∗∗^ *p* < 0.001. NS: no significance.

The inflammation response to AMI *in vivo* was measured by detecting the infiltration of inflammatory cells by flow cytometry. Here, we screened different kinds of immune cells, such as B cells, T cells, natural killer cells, neutrophils, and monocytes/macrophages, in both heart tissues and whole blood. We found that the infiltrated macrophages and neutrophils significantly increased in the heart tissues that suffered from AMI, while the other types of immune cells were not detected in the heart tissues. There were more infiltrated macrophages and neutrophils in the hearts in SOD1^−/−^ mice than that in WT mice after AMI (Figures 3(a)–3(c)). This was confirmed by immunohistochemistry of CD11b (Figure 3(d)). Moreover, the inflammatory cytokines IL-6 and TNF-*α* in the heart tissues and blood samples were detected by ELISA assay. The level of IL-6 and TNF-*α* increased significantly in the ischemic heart tissues, and they increased further in SOD1 KO mice subjected to AMI (Figures 3(e) and 3(f)). The levels of blood IL-6 and TNF-*α* among the different groups were not significantly different. The concentration of IL-6 in the plasma was approximately 35 ± 15 pg/mL, and TNF-*α* was barely detectable. There were no differences in the percentage of neutrophils and monocytes in the blood among different groups (Figures 3(g) and 3(h)). This indicates that the inflammation response in the ischemic myocardium was regional, not systemic. The above results suggest that cardiac injury was enhanced in AMI mouse models under excessive oxidative stress.

### 3.2. Oxidative Stress Aggravated Apoptosis and Induced Enhanced Autophagy in Cardiomyocytes

**Figure 4:**
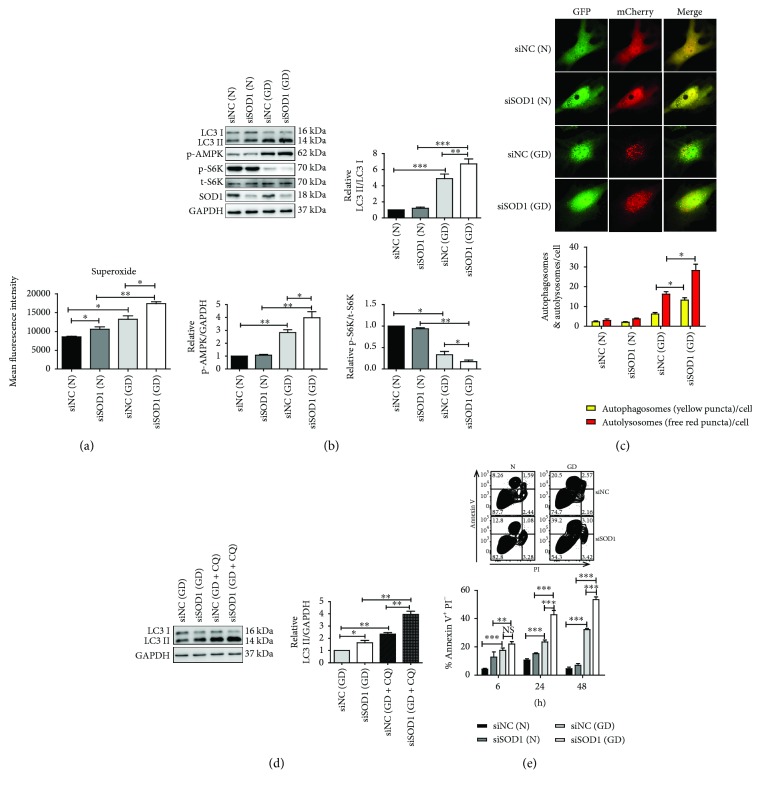
Autophagy and apoptosis levels increased in SOD1-knockdown neonatal rat cardiomyocytes under glucose deprivation. (a) Quantitative analysis of superoxide levels in NRCMs. (b) Representative blots and quantitative analysis of LC3II/LC3I and S6K phosphorylation and AMPK phosphorylation. (c) Representative images and quantitative analysis of autophagy flux examined by fluorescent mCherry-GFP-LC3 signal (original magnification, ×400). Yellow puncta represent autophagosomes. Free red puncta represent autolysosomes. (d) Autophagy flux in glucose-deprived NRCMs was inhibited by 10 *μ*M chloroquine. Representative blots and quantitative analysis of LC3II/GAPDH are shown. (e) Representative FACS results at 24 h and quantitative analysis of apoptosis levels from 6 to 48 h are shown. Values represent mean ± SE of three independent experiments. ^∗^ *p* < 0.05, ^∗∗^ *p* < 0.01, and ^∗∗∗^ *p* < 0.001. N: normal condition; GD: glucose deprivation.

To detect the effect of oxidative stress on cardiomyocytes *in vitro*, the apoptosis and autophagy levels were examined in SOD1 knockdown NRCMs. Here, glucose deprivation (GD) was used to mimic *in vivo* myocardial ischemia conditions. First, superoxide level was detected by DHE staining with flow cytometry. The superoxide level in SOD1 knockdown NRCMs significantly increased. GD induced higher superoxide levels and LC3II/LC3I ratios in NRCMs compared to that in control conditions (Figures 4(a) and 4(b)). Furthermore, the GD-induced LC3II/LC3I ratio was significantly increased by SOD1 knockdown (Figure 4(b)). GD increased AMPK phosphorylation that was further increased by SOD1 knockdown. Moreover, the level of S6K phosphorylation was significantly inhibited under GD, especially in siSOD1-transfected NRCMs (Figure 4(b)).

Additionally, we monitored the autophagy flux by using mCherry-GFP-LC3 adenovirus. The results showed that GD significantly increased the number of autophagosomes (yellow puncta) and autolysosomes (free red puncta) in both siNC- or siSOD1-treated NRCMs. SOD1 knockdown stimulates further increase in autophagosomes and autolysosomes under GD (Figure 4(c)). The autophagy flux was also measured by adding 10 *μ*M CQ to inhibit lysosome-mediated proteolysis. The results showed that LC3-II protein levels increased in both siNC- and siSOD1-treated NRCMs in the presence of CQ. LC3-II increased further in SOD1-knockdown NRCMs in the presence of CQ under GD (Figure 4(d)). These results suggested that the oxidative stress in NRCMs induced by SOD1 knockdown increased the autophagy flux under GD. Furthermore, apoptosis levels in NRCMs under GD were measured. The results showed that there was no difference between siNC- and siSOD1-treated NRCMs under normal conditions from 6 to 48 h. However, the number of apoptotic cells significantly increased in siSOD1-treated NRCMs compared to that in siNC-treated NRCMs under GD from 24 to 48 h (Figure 4(e)). This demonstrated that oxidative stress in NRCMs increased apoptosis and autophagy *in vitro* under GD.

**Figure 5:**
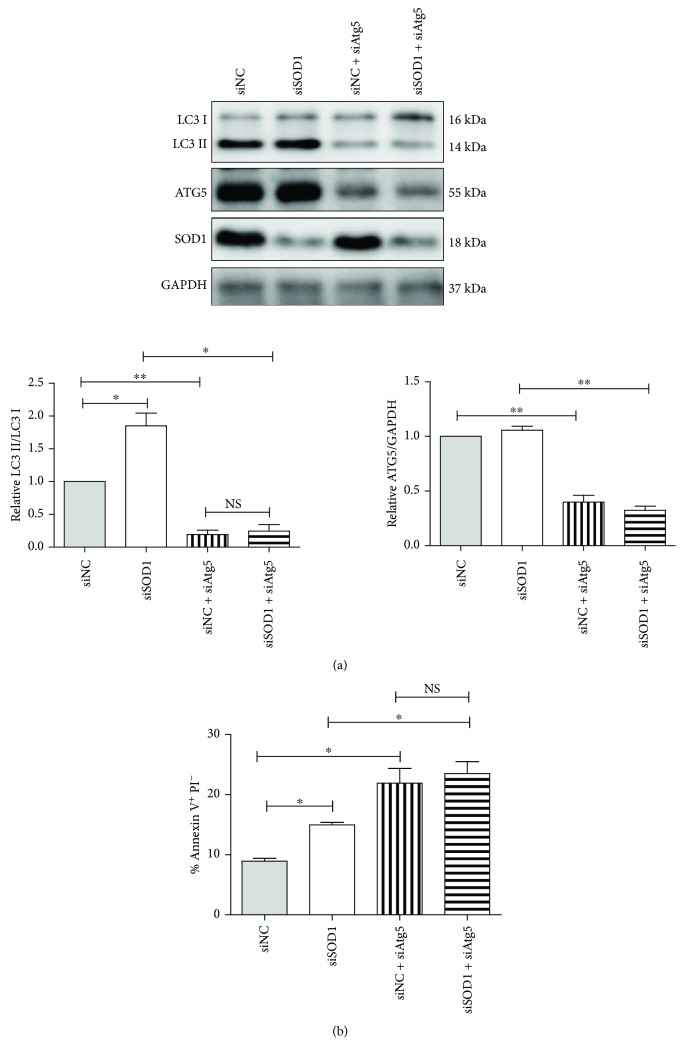
Autophagy and apoptosis levels in Atg5-knockdown NRCMs under glucose deprivation. (a) Representative blots and quantitative analysis of LC3II/LC3I and ATG5. (b) Quantitative analysis of apoptosis levels among different groups at 24 h under GD. Values represent mean ± SE of three independent experiments. ^∗^ *p* < 0.05 and ^∗∗^ *p* < 0.01. NS: no significance.

To investigate the relationship between autophagy and apoptosis under excessive oxidative stress, we blocked autophagy using Atg5 siRNA. The results showed that knockdown of Atg5 significantly inhibited autophagy, but enhanced apoptosis, in siNC- or siSOD1-treated NRCMs under GD. SOD1 knockdown had no effect on autophagy and apoptosis in Atg5 knockdown NRCMs under GD (Figure 5). This indicates that autophagy protects cells from apoptotic cell death under oxidative stress.

### 3.3. NaHS Alleviated GD-Induced Apoptosis and Autophagy in NRCMs under Excessive Oxidative Stress *In Vitro*

**Figure 6:**
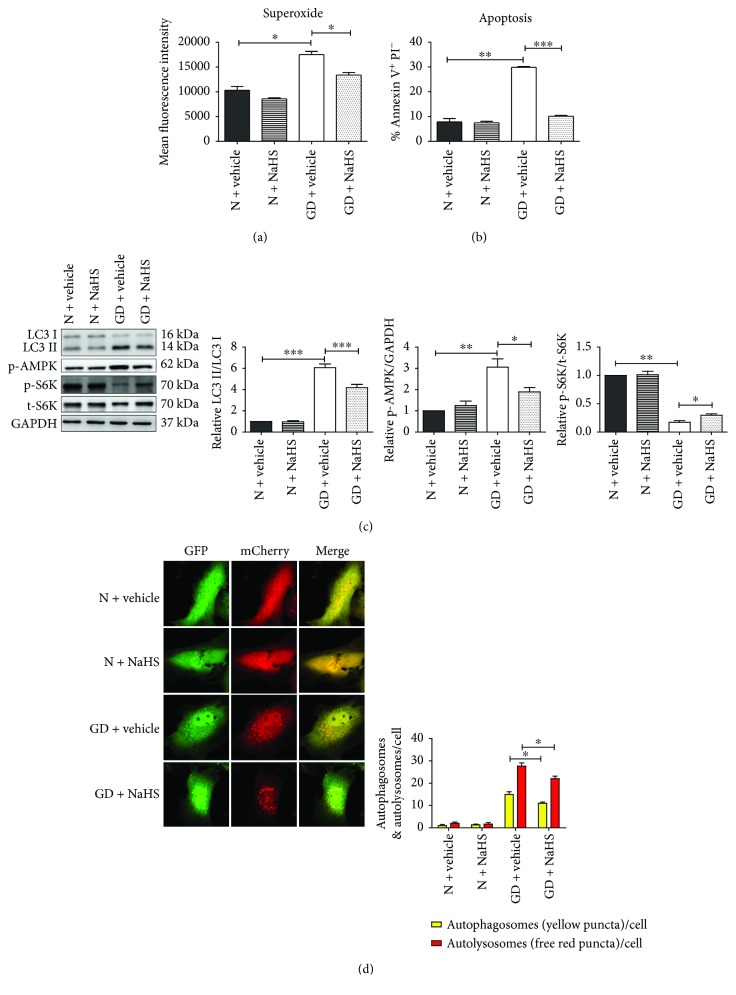
NaHS treatment reduced autophagy and apoptosis induced by glucose deprivation in SOD1-knockdown neonatal rat cardiomyocytes. (a) Quantitative analysis of superoxide levels in SOD1-knockdown NRCMs under normal conditions or GD with or without 50 *μ*M NaHS treatment. (b) Quantitative analysis of apoptosis levels in SOD1-knockdown NRCMs with or without NaHS treatment for 24 h. (c) Representative blots and quantitative analysis of LC3II/LC3I and S6K phosphorylation and AMPK phosphorylation after SOD1 knockdown in NRCMs with or without NaHS treatment. (d) Representative images and quantitative analysis of autophagy flux examined by fluorescent mCherry-GFP-LC3 signal (original magnification, ×400). Yellow puncta represent autophagosomes. Free red puncta represent autolysosomes. Values represent mean ± SE of three independent experiments. ^∗^ *p* < 0.05, ^∗∗^ *p* < 0.01, and ^∗∗∗^ *p* < 0.001.

H_2_S is a bioactive small molecule with reducibility and plays important roles in regulating cardiovascular diseases. In this study, 50 *μ*M NaHS was used to treat SOD1-knockdown NRCMs. As shown in Figure 6(a), the superoxide levels significantly increased in siSOD1-treated NRCMs under GD, but 30 min of pretreatment with NaHS significantly decreased the superoxide levels in siSOD1-treated NRCMs under GD. NaHS pretreatment significantly reduced the apoptosis levels in siSOD1-treated NRCMs under GD (Figure 6(b)). Furthermore, LC3II/LC3I decreased in SOD1-knockdown NRCMs pretreated with NaHS under GD (Figure 6(c)). The data showed that NaHS treatment partially inhibited AMPK and activated mTOR activity in siSOD1-treated NRCMs under GD (Figure 6(c)). By monitoring the autophagy process with mCherry-GFP-LC3 adenovirus, we found that NaHS pretreatment reduced the number of autophagosomes and autolysosomes under GD in SOD1-knockdown NRCMs (Figure 6(d)). This revealed that NaHS pretreatment can reduce apoptosis and autophagy levels in siSOD1-treated NRCMs under GD.

### 3.4. NaHS Relieved the AMI-Induced Cardiac Injury under Oxidative Stress *In Vivo*

**Figure 7:**
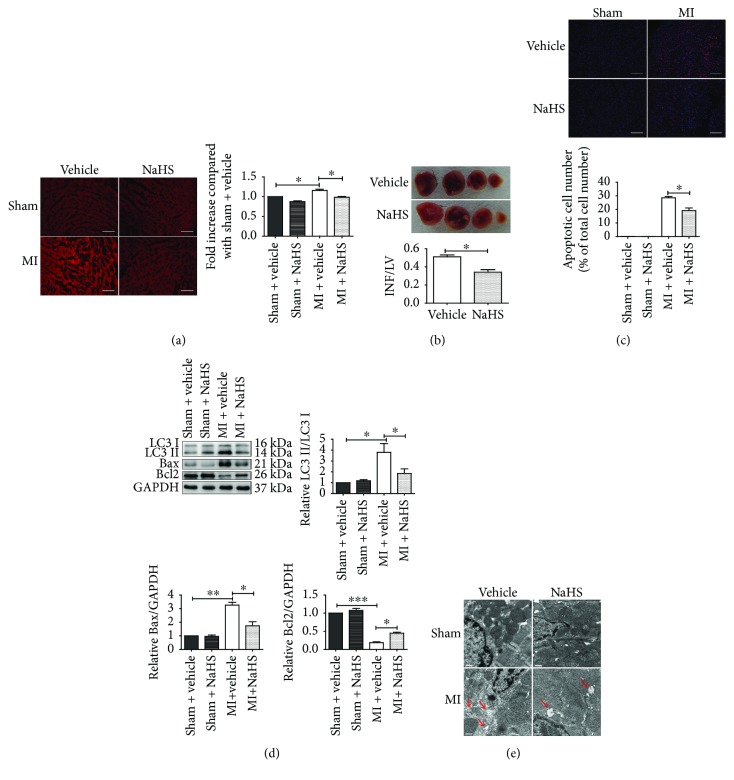
NaHS treatment alleviated apoptosis and autophagy in the ischemic myocardium from SOD1 KO mice. (a) Representative images and quantitative analysis of superoxide levels in SOD1 KO mice after AMI surgery with or without 50 *μ*mol/kg NaHS treatment. Scale bar = 50 *μ*m. (b) Representative images and quantitative analysis of the infarct size with or without NaHS treatment. The white area represents INF. LV: left ventricle; INF: infarct area. (c) Representative images and quantitative analysis of apoptotic cells in the myocardium of SOD1 KO mice after AMI surgery with or without NaHS treatment. TUNEL staining is shown in red. Nuclei are shown in blue. Scale bar = 50 *μ*m. (d) Representative blots and quantitative analysis of LC3II/LC3I, Bax, and Bcl2. (e) Representative images of TEM results are shown. Red arrows indicate autophagic vacuoles. Scale bar = 0.5 *μ*m. Values are mean ± SE, *n* = 6 in each group. ^∗^ *p* < 0.05, ^∗∗^ *p* < 0.01, and ^∗∗∗^ *p* < 0.001.

To determine whether NaHS protects SOD1^−/−^ mice after AMI, SOD1^−/−^ mice were treated with saline or NaHS 30 min before surgery. NaHS pretreatment significantly decreased the superoxide level in the ischemic myocardium compared to saline pretreatment (Figure 7(a)). TTC staining showed that NaHS pretreatment decreased the infarct size of SOD1^−/−^ mice (Figure 7(b)). TUNEL assay showed that NaHS pretreatment significantly decreased the percentage of apoptotic cells in the infarct area (Figure 7(c)). Furthermore, NaHS pretreatment reduced the expression of Bax, increased the expression of Bcl2, and reduced the LC3II/LC3I ratio, in the ischemic myocardium in SOD1^−/−^ mice (Figure 7(d)). The morphological studies showed that NaHS pretreatment reduced the autophagic vacuoles in the ischemic myocardium in SOD1^−/−^ mice (Figure 7(e)). This suggested that NaHS pretreatment could reduce the autophagy level in the ischemic heart tissues in SOD1^−/−^ mice.

**Figure 8:**
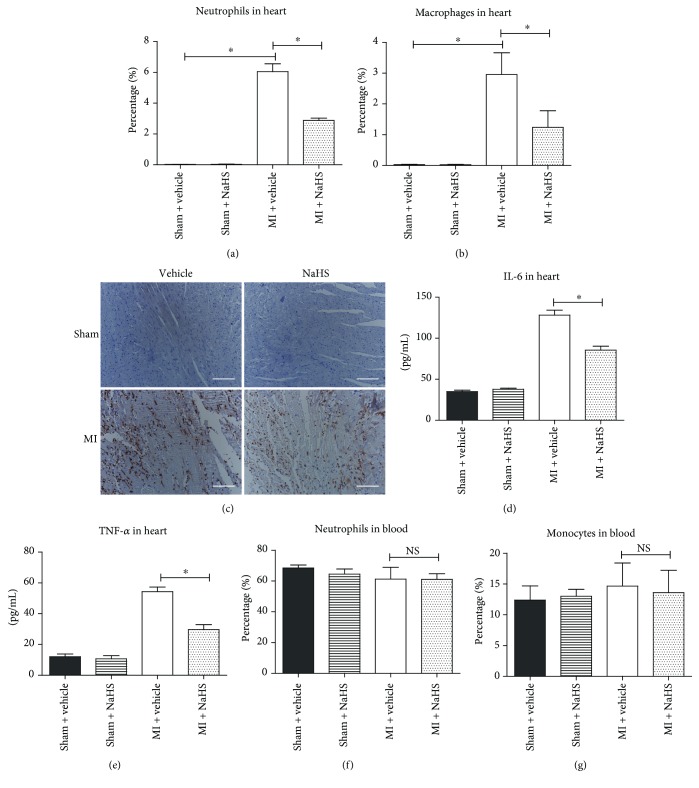
Exogenous H_2_S alleviated inflammation response in ischemic myocardium from SOD1 KO mice. (a–b) Quantitative analysis of neutrophils (a) and macrophages (b) from SOD1 KO heart samples. (c) Immunohistochemistry of CD11b from SOD1 KO mice. Positive staining is shown in brown. Scale bar = 50 *μ*m. (d–e) Quantitative analysis of inflammatory cytokines IL-6 (d) and TNF-*α* (e) in heart tissues. (f–g) Quantitative analysis of neutrophils (f) and monocytes (g) in blood samples from SOD1 KO mice. Values are mean ± SE, *n* = 6 in each group. ^∗^ *p* < 0.05. NS: no significance.

To test the effect of NaHS treatment on the regional inflammation response in the heart tissues of SOD1^−/−^ mice, the infiltrated immune cells were detected by flow cytometry. The data showed that NaHS (pretreatment for 30 min and persisting for 2 d after AMI) decreased the amounts of macrophages and neutrophils in the ischemic myocardium in SOD1^−/−^ mice compared to saline controls (Figures 8(a) and 8(b)). This was confirmed by immunohistochemistry of CD11b (Figure 8(c)). Moreover, the ELISA assay showed that NaHS could reduce the inflammatory cytokines IL-6 and TNF-*α* in the ischemic heart tissues in SOD1^−/−^ mice (Figures 8(d) and 8(e)). This demonstrated that NaHS relieved the regional inflammation response in AMI heart tissues under oxidative conditions *in vivo*. Moreover, NaHS pretreatment did not affect the amounts of blood monocytes and neutrophils in SOD1^−/−^ mice (Figures 8(f) and 8(g)) or the levels of IL-6 and TNF-*α* in plasma (data not shown). Taken together, these data suggest that NaHS pretreatment alleviated the AMI-induced cardiac injury under excessive oxidative stress *in vivo*.

## 4. Discussion

In the present study, we investigated the effect of excessive oxidative stress on cardiac injury during AMI and the role of H_2_S in regulating this process in both an *in vivo* AMI model and in an *in vitro* GD-mimic MI model. The experiments demonstrated the following important findings: (1) autophagy and apoptosis levels markedly increased during myocardial ischemia under excessive oxidative stress both *in vitro* and *in vivo*; (2) the regional inflammation response was more serious in SOD1^−/−^ mice after AMI; (3) NaHS pretreatment decreased autophagy and apoptosis in the ischemic myocardium under oxidative stress both *in vivo* and *in vitro*; and (4) H_2_S alleviated the inflammation response in ischemic heart tissues under oxidative stress *in vivo*.

Accumulating evidence has indicated that excessive ROS was produced during myocardial ischemia, thus leading to oxidative stress in the heart [46, 47]. However, it is not clear whether people with high ROS levels are susceptible to AMI. In this study, we used SOD1^−/−^ mice to study the effect of excessive oxidative stress on cardiac injury during AMI and the role of exogenous H_2_S in this process. We found that higher ROS (superoxide) levels in tissue usually correlated with larger infarct size. Moreover, excessive oxidative stress significantly enhanced the apoptosis levels in ischemic cardiomyocytes during AMI. Our results demonstrated that autophagy levels increased significantly in AMI heart tissues, especially in SOD1 KO mice, protecting cells from apoptotic cell death. Excessive oxidative stress markedly increased the inflammation response in the ischemic heart tissue, indicating that SOD1^−/−^ mice were more vulnerable to AMI.

In addition, our results demonstrated that AMPK was activated while mTOR activity was not reduced during AMI. This was in agreement with a previous report by Matsui et al. [26]. Furthermore, our results showed that the activity of AMPK was increased further under excessive oxidative stress. This suggested that the excessive oxidative stress may induce enhanced autophagy by activating AMPK. Matsui et al. reported that increased autophagy was protective during ischemia [26]. We found that knockdown of the essential autophagy protein ATG5 in siNC- or siSOD1-treated NRCMs under GD significantly increased the apoptosis levels. This suggested that enhanced autophagy is protective under GD. It is generally believed that autophagy is usually prosurvival; it allows cells to survive prolonged starvation, injury, and other stresses [48]. Our results showed that knockdown of Atg5 blocked further increase in apoptosis level in siSOD1 treated NRCMs under GD. Damaged cells in heart tissues trigger the inflammation response [49]. Enhanced inflammation response may lead to increased secretion of inflammatory cytokines such as TNF-*α* and IL-6. It was suggested that high levels of TNF-*α* or IL-6 may promote cardiomyocyte apoptosis [50, 51]. Finally, excessive oxidative stress resulted in aggravated cardiac injury. The exact mechanism of the interplay among autophagy, apoptosis, and inflammation response under oxidative stress still needs further study.

Previous studies utilized GD in cultured cardiomyocytes to mimic myocardial ischemia *in vivo* [26, 52]. In this study, we induced oxidative stress by SOD1 knockdown in cardiomyocytes, which increased apoptosis and autophagy. This is consistent with our results *in vivo*. Autophagy is modulated by mTOR and AMPK by various mechanisms [53]. Our results showed that GD stimulated activation of AMPK and inactivation of mTOR and induced autophagy, consistent with a previous report by Matsui et al. [26]. Additionally, we found that excessive oxidative stress enhanced autophagy levels by modulating the AMPK-mTOR pathway. It was reported that ROS can regulate autophagy by affecting the autophagy-related gene 4 (ATG4) [54]. Tang et al reported that ROS can induce autophagy by targeting high mobility group box 1 (HMGB1) [55]. However, the precise mechanism by which oxidative stress regulates autophagy via modulating the AMPK-mTOR pathway needs further study.

Accumulating evidence has confirmed that H_2_S is a gasotransmitter that plays important roles in various physiologic processes, especially in the cardiovascular system. It is reported that H_2_S protects cardiomyocytes from myocardial ischemia-reperfusion injury [42, 56–59]. We observed that H_2_S protected cardiomyocytes from AMI injury by reducing autophagy activity and apoptosis levels, consistent with previous reports. Interestingly, we reported here for the first time that H_2_S can reduce autophagy and apoptosis levels during AMI with the exacerbated background oxidative stress (in SOD1 knockdown NRCMs and SOD1^−/−^ mice). However, the precise mechanism by which H_2_S reduces autophagy levels via regulating the AMPK-mTOR pathway under oxidative stress needs further investigation.

Recent studies indicate that H_2_S played a critical role in inflammation response in various diseases [60]. In this study, we demonstrated that H_2_S reduced regional inflammatory cell infiltration towards the infarcted area in heart tissues. We also found that the inflammation response in the infarcted hearts was regional but not systemic. NaHS treatment can reduce inflammation response under oxidative stress. Our study extends the knowledge that H_2_S acts as an anti-inflammation molecule in the cardiovascular system. However, the exact mechanism by which H_2_S alleviates the inflammation response by reducing the infiltration of immune cells and the level of inflammatory cytokines remains unknown. Taken together, our study presents evidence that exogenous H_2_S can protect cardiomyocytes from AMI injury by reducing excessive apoptosis and inflammation response under oxidative stress in SOD1^−/−^ mice.

## 5. Conclusion

In conclusion, our present findings support the idea that individuals with high ROS levels are susceptible to AMI. Both autophagy and apoptosis levels were elevated, and the inflammation response in ischemic heart tissues was increased during AMI with exacerbated background oxidative stress. Increased apoptosis and autophagy levels were reversed by H_2_S supplementation. Exogenous H_2_S played a vital role in protecting cardiomyocytes from AMI injury by mitigating excess inflammation response in the ischemic heart tissues.

## Supplementary Materials

Supplementary materials Supplemental materials and methods: the designed SOD1-targeting siRNA sequences were 5′-GCATGGGTTCCATGTCCATCA-3′ (siSOD1-1) and 5′-GGTGGTCCACGAGAAACAAGA-3′ (siSOD1-2). The two targeting siRNA sequences and the negative control siRNA (siNC) were synthesized by Biotend (Shanghai, China). After culturing for 48 h, NRCMs were transfected with 40 pmol siRNA per 6-well plate using Lipofectamine RNAiMAX (Invitrogen, Carlsbad, CA, USA) per the manufacturer's instructions. The knockdown efficiency was detected by Western blotting 48 h after transfection. Supplemental results: to avoid the off-target effect, two SOD1-targeting siRNAs were tested. The Western blotting results showed that the two SOD1-targeting siRNAs were both effective, with approximately 70% knockdown efficiency (Supplemental Figure S1). Therefore, one of the two SOD1-targeting siRNAs was used in the following experiments (siSOD1-2) and named siSOD1. Supplemental Figure S1: representative blots and quantitative analysis of SOD1 expression after siRNA knockdown. Values represent mean ± SE of three independent experiments. ^∗∗^ *p* < 0.01.
